# Reduced susceptibility of tomato stem to the necrotrophic fungus *Botrytis cinerea* is associated with a specific adjustment of fructose content in the host sugar pool

**DOI:** 10.1093/aob/mcw240

**Published:** 2017-01-08

**Authors:** François Lecompte, Philippe C. Nicot, Julie Ripoll, Manzoor A. Abro, Astrid K. Raimbault, Félicie Lopez-Lauri, Nadia Bertin

**Affiliations:** 1 PSH unit, INRA, F-84914 Avignon, France; 2 Plant pathology unit, INRA, F-84140 Montfavet, France; 3 UMR Qualisud, Université d’Avignon et des Pays du Vaucluse, F-84916 Avignon, France

**Keywords:** Tomato (*Solanum lycopersicum*), *Botrytis cinerea*, necrotrophic fungi, plant defence, soluble sugars, fructose, glucose, hormones

## Abstract

**Background and aims** Plant soluble sugars, as main components of primary metabolism, are thought to be implicated in defence against pathogenic fungi. However, the function of sucrose and hexoses remains unclear. This study aimed to identify robust patterns in the dynamics of soluble sugars in sink tissues of tomato plants during the course of infection by the necrotrophic fungus *Botrytis cinerea*. Distinct roles for glucose and fructose in defence against *B. cinerea* were hypothesized.

**Methods** We examined sugar contents and defence hormonal markers in tomato stem tissues before and after infection by *B. cinerea*, in a range of abiotic environments created by various nitrogen and water supplies.

**Key Results** Limited nitrogen or water supplies increased tomato stem susceptibility to *B. cinerea*. Glucose and fructose contents of tissues surrounding infection sites evolved differently after inoculation. The fructose content never decreased after inoculation with *B. cinerea*, while that of glucose showed either positive or negative variation, depending on the abiotic environment. An increase in the relative fructose content (defined as the proportion of fructose in the soluble sugar pool) was observed in the absence of glucose accumulation and was associated with lower susceptibility. A lower expression of the salicylic acid marker *PR1a*, and a lower repression of a jasmonate marker *COI1* were associated with reduced susceptibility. Accordingly, COI1 expression was positively correlated with the relative fructose contents 7 d after infection.

**Conclusions** Small variations of fructose content among the sugar pool are unlikely to affect intrinsic pathogen growth. Our results highlight distinct use of host glucose and fructose after infection by *B. cinerea* and suggest strongly that adjustment of the relative fructose content is required for enhanced plant defence.

## INTRODUCTION

Upon infection by fungal pathogens, plants induce a massive reprograming of their genome expression and metabolism to set up a range of defence reactions. Although some mechanisms are universally activated in response to biotic stresses, the integrated immune response of the host plant is specific, as a consequence of the range of virulence factors that each pathogen species can produce according to its lifestyle (Glazebrook, 2005; Mengiste, 2012). Pathogenic fungi and oomycetes are traditionally classified according to the way they colonize and feed from their hosts: biotrophs carry out extracellular colonization of living host tissues and develop specialized structures to penetrate cell walls and retrieve intracellular nutrients, while necrotrophs destroy both intercellular and cellular structures by releasing cell-wall-degrading enzymes and toxic compounds in order to feed from dead tissues. An intermediate lifestyle, hemibiotrophy, entails the succession of a biotrophic and a necrotrophic phase in the infection process. Although this classification might appear an oversimplification of virulence strategies of pathogenic fungi (Oliver and Solomon, 2010; Van Kan *et al.*, 2014), it coincides with the prominent antagonism between hormonal signalling of salicylic acid (SA) and jasmonate (JA), which trigger systemic responses against biotrophic and necrotrophic fungi, respectively. Beside these two major pathways, the fine tuning of signalization in the plant’s immune system relies on a complex network involving most plant hormones, including abscissic acid (ABA), ethylene (ET) and gibberellic acid (GA), presumably to allocate resources for growth, reproduction and defence in a cost-efficient manner (Pieterse *et al.*, 2012; Yang *et al.*, 2012; Huot *et al.*, 2014).

The modification by the plant of its primary metabolism upon infection by a pathogen has been frequently reported for both biotrophic and necrotrophic interactions (Berger *et al.*, 2007; Bolton, 2009; Rojas *et al.*, 2014). Differential expression of primary metabolism genes in plants infected by the necrotroph *Botrytis cinerea* has been shown in several studies (Birkenbihl *et al.*, 2012; Windram *et al.*, 2012; De Cremer *et al.*, 2013; Vega *et al.*, 2015). At the basis of this central metabolism of the host plant, the main soluble sugars sucrose, glucose and fructose are involved in plant–pathogen interactions in multiple ways (Moghaddam and Van den Ende, 2012; Morkunas and Ratajczak, 2014; Rojas *et al.*, 2014). The catabolism of soluble sugars is considered to be the primary source of carbon and energy for the production of a range of secondary metabolites and of C-based polymers for strengthening the cell wall (Gershenzon, 1994; Heil and Baldwin, 2002; Koricheva, 2002; Swarbrick *et al.*, 2006; Berger *et al.*, 2007). In source tissues such as leaves, infection by *B. cinerea* induces the repression of genes involved in photosynthesis (Berger *et al.*, 2004; Bilgin *et al.*, 2010; Windram *et al.*, 2012; De Cremer *et al.*, 2013) and the activation of extracellular invertases, resulting in an accumulation of hexoses and a decrease in the sucrose/hexose ratio in infected tissues (Berger *et al.*, 2004). On Arabidopsis and lettuce leaves, infection was shown to enhance the expression of most genes involved in sucrose catabolism, glycolysis, the oxidative pentose phosphate pathway and the TCA cycle (De Cremer *et al.*, 2013; Ferrari *et al.*, 2007). In tomato leaves, infection repressed glycolysis while an overexpression of some genes involved in the TCA pathway was observed (Vega *et al.*, 2015). Genes coding for sugar transporters are also up-regulated, suggesting coordination between sucrose cleavage by invertases and cellular hexose uptake (Fotopoulos *et al.*, 2003; Lemonnier *et al.*, 2014). Additionally, during compatible reactions, either with biotrophic or necrotrophic fungi, a flux of hexose from the host cells to the pathogen is set up, via the activation of hexose transporters of fungal origin (Dulermo *et al.*, 2009; Voegele *et al.*, 2001). Plant sugar efflux transporters from the SWEET family are also involved and can be a target of pathogen effectors, possibly to facilitate sugar uptake by the pathogen (Chen *et al.*, 2010). Genes coding for some SWEET transporters are activated following *B*. *cinerea* infections in Arabidopsis and grapevine (Ferrari *et al.*, 2007; Chong *et al.*, 2014). However, their role in pathogenicity remains unclear. The involvement of sugars in defence goes largely beyond their involvement in primary metabolic pathways. Sugars, and in some cases hexose kinases, are involved in multiple steps of the regulation of host immunity, including the activation of pathogenesis-related proteins (PR) (Herbers *et al.*, 1996; Salzman *et al.*, 1998; Thibaud *et al.*, 2004; Essmann *et al.*, 2008; Kocal *et al.*, 2008), the modulation of the transcription of enzymes involved in the biosynthesis of secondary metabolites (Herbers *et al.*, 1996; Solfanelli *et al.*, 2006; Ferri *et al.*, 2011; Morkunas *et al.*, 2011) or the regulation of the cell redox status and production of reactive oxygen species (Kim *et al.*, 2006; Essmann *et al.*, 2008).

Despite the numerous studies implicating soluble sugars in plant defence, the specific roles of individual sugars and their possible coordination or antagonism remain to be elucidated. There is compelling evidence, since the pioneering work of Horsfall and Dimond (1957), that the pathogen’s lifestyle determines the empirical relationship between host sugar content and disease severity. Part of this evidence stems from studies with plants grown in conditions of low light intensity. Fungi with a necrotrophic phase have been reported to develop better on plants growing under low light intensity, or under light with a low red/far red ratio (Thomas and Allen, 1971; Victoria and Thurston, 1974; Hammer and Evensen, 1994; Garcia-Guzman and Heil, 2014). Low red/far red ratio has been shown to repress JA-dependent defence responses (Cerrudo *et al.*, 2012; Chico *et al.*, 2014; Garcia-Guzman and Heil, 2014). It was further shown recently that JA-deficient mutants of *Nicotiana attenuata* present an increased activity of intracellular invertases, suggesting a link between hormonal defence signals and primary metabolic status (Machado *et al.*, 2015). However, low light levels can also alter the soluble sugar content of plant tissues. Limited sugar availability might restrict the catabolism of sucrose and hexoses needed for fuelling plant defence and maintaining host cell integrity, the latter being hypothesized as a possible defence strategy against necrotrophic fungi (Seifi *et al.*, 2013). However, the host range of necrotrophic fungi such as *B. cinerea* and *Sclerotinia sclerotiorum* is known to encompass hundreds of plants species and organs (Elad *et al.*, 2016), and the literature lacks general trends showing that plant species or plant organs containing high carbohydrate levels may be either more resistant (because of enhanced defence) or conversely susceptible (because of higher sugar availability for the pathogen) to necrotrophic fungi. Carbohydrate availability in Arabidopsis leaves during the early necrotrophic phase of the hemibiotroph *Colletotrichum higginsianum* influences host susceptibility (Engelsdorf *et al.*, 2013). Indeed, low total carbohydrate availability (main sugars plus starch) was shown to promote necrotrophic fungal invasion, presumably by perturbing host defences, while a lower carbon availability *per se* did not affect fungal growth. On the contrary, some biotrophic fungi grow slower when the carbon availability in host cells is reduced (Engelsdorf *et al.*, 2013). As mentioned above, there is much evidence that, for intact or detached source tissues such as leaves, resistance to a range of both biotrophic and necrotrophic pathogens requires the depolymerization of starch as well as the use of sucrose to produce glucose and fructose via the activity of invertases and possibly sucrose synthase. In recent work, we observed in leaf tissues of lettuce that the relative contents of fructose and sucrose at the time of infection by *B. cinerea* and *S. sclerotiorum* were strongly correlated with the resulting symptoms, while the absolute or relative content of glucose was apparently not correlated with disease severity (Lecompte *et al.*, 2013).

These considerations led to the hypothesis that glucose and fructose might not play an equivalent role in plant defence against necrotrophs, and that the adjustment of specific sugar balances might be a determinant of susceptibility. In the present study we modulated the nitrogen or water supply during plant growth to achieve different levels of soluble sugars in the tissues of tomato plants and subjected them to inoculation with *B. cinerea*. Tomato stems are vulnerable to this pathogen, as a consequence of leaf pruning which is a common practice in greenhouse cultivation (O’Neill *et al.*, 1997; Shtienberg *et al.*, 1998). Compiling results of several experiments, we identified the relative fructose content (RFC), defined as the proportion of fructose in the pool of sucrose, glucose and fructose, as a major marker of tomato stem defence to *B*. *cinerea*. We show differential adjustments of glucose and fructose in host tissues surrounding lesions, and suggest that fructose must be maintained in a specific concentration range in the soluble sugar pool for efficient defence. We present evidence of concomitant evolution of sugar contents and markers of hormonal defence signals during the course of infection.

## MATERIALS AND METHODS

### Experimental layout

Eight glasshouse experiments were carried out between 2010 and 2014. The plants were subjected to different fertigation regimes for several weeks prior to their inoculation with *B. cinerea* and incubated under controlled conditions in a growth chamber. In the first five experiments (E1 to E5), tomato plants (‘Swanson’) were fertigated with five nutrient solutions differing in their nitrate concentrations for 4 weeks prior to inoculation. These experiments were used to examine the relationship between the plant’s primary metabolic status at the time of inoculation and the severity of symptoms. Bioassays on detached leaves were also performed in E3 and E5. In experiment E6, the findings of previous experiments were validated for two levels of nitrate fertigation on a different accession of tomato (‘Momor’). Additionally, the dynamics of primary metabolites was monitored in the stems after inoculation with *B. cinerea*, in parallel with disease development. Experiments E7 and E8 were designed to validate results with plants grown with contrasted water supplies. In E7, tomato plants (‘Momor’ and ‘Monalbo’) were grown under three regimes of water supply for 3 weeks. After inoculation, the dynamics of primary metabolites was monitored, as in E6. Additionally, levels of SA and JA signalling markers and stem ABA contents were measured. Bioassays on detached leaves were performed. Experiment E8 was a repetition of the bioassays on leaves and stems done in E7, with similar procedures and the same genotypes.

The period of the year when plants were grown in the glasshouse was not identical for all experiments, varying between February and November. Cumulated global irradiance during the 10-d period before plant inoculation exceeded 15000 J cm^−2^ in all but one experiment, E6, which was conducted in autumn with lower natural irradiance (5740 J cm^−2^ during the 10-d period preceding inoculation).

### Plant production, N and water treatments

Plants were produced and fertigated in E1 to E6 as described in Lecompte *et al.* (2010). Briefly, 10-d-old plantlets were transferred onto rock wool blocks 7·5 × 7·5 × 6 cm (Grodan, Roermond, the Netherlands). During the first month, the plants were fertigated twice a day with a standard commercial nutrient solution (Duclos International, Lunel-Viel, France). After that period, the plants (bearing 3–4 leaves) were placed on the top of 2-L pots filled with a mixture (1^:^1, v/v) of vermiculite and pozzolana to start the nutrition treatments. Three blocks of ten plants per nutrition treatment were positioned at random in the glasshouse. In E1 to E5, five different nutrient solutions were used, containing 0·5 mm${\text{NO}}_{3}^{-}$, 2 mm${\text{NO}}_{3}^{-}$, 5 mm${\text{NO}}_{3}^{-}$, 10 mm${\text{NO}}_{3}^{-}$ and 20 mm${\text{NO}}_{3}^{-}$. In E6, only two nitrate concentrations (2 and 15 mm) were retained. The equilibrium in electric charges was maintained by replacing nitrates with sulphates in the solutions with lower nitrate concentrations. The concentration of other major nutrient elements was kept constant, at the following levels: 11 mm K, 3·5 mm Mg, 3·5 mm Ca and 1 mm P. Micronutrients were present at the following concentrations (in µmol L^−1^): 20·6 B, 0·5 Cu, 10·7 Fe, 11·6 Mn, 0·28 Mo and 3·2 Zn. The plants were fertigated with a drip irrigation system up to six times a day depending on the climatic demand, with 1-min pulses. Three pots chosen at random were weighed continuously to evaluate their actual evapotranspiration and water demand. The pH was adjusted to 6 in each treatment by addition of H_2_SO_4_. Plants were grown with those solutions for 4 weeks before inoculation.

In E7 and E8, 1-month old plantlets were transferred to 4-L pots filled with a commercial horticultural substrate composed of white peat and compost with 1 g L^−1^ of 14-10-18 NPK fertilizer (TS 3 substrate N°404, Klasmann, Champety, France). Plants were drip-irrigated daily with a commercial fertilizer (Fertiplant 16 10 24, Plantin, Courthézon, France). Irrigation was based on continuous weighing of soil and plants at drainage. Treatments started 45 (± 1) d after sowing. Three blocks of ten plants for each irrigation × genotype combination were arranged at random in the glasshouse. The three fertigation treatments were a control (CO) with irrigation compensating for actual evapotranspiration, and two levels of decreased water supply, consisting of a 60 and an 80 % reduction of the water provided in the control (WS60 and WS80, respectively). Nutrient concentrations in the irrigation solutions were adjusted so that plants in the different treatments received the same amount of fertilizers. Drainage of CO irrigation averaged 25 % throughout the experiments while it was close to 0 in WS60 and WS80. However in all the treatments the EC range was 0·7–1 mS cm^−1^, and salinity did not increase significantly in the substrate of plants under water stress. The effects of water treatments were assessed by measuring substrate relative humidity (WCM Control; Grodan) and stem water potentials at predawn and midday on non-senescent mature leaves (pressure chamber SAM Précis 2000; Gradignan, France). Plant inoculation was carried out at 65 (± 1) d after sowing. At that time, in E7, substrate relative humidity was 69, 42 and 32 % and midday water potential was −0·38, −0·46 and −0·55 MPa in CO, WS60 and WS80, respectively. Comparable values were recorded in E8.

### Inoculation of leaf pruning wounds with conidia of *B. cinerea*

Inoculations were carried out in the morning (between 0900 and 1000 h) on five plants for each genotype × fertigation treatment combination. Leaves were removed from each plant, leaving 5-mm petiole stubs on the stems, and a 10-μL aliquot of *B. cinerea* conidia suspension was immediately deposited on each pruning wound. Conidia of *B. cinerea* (strain BC1 in all experiments, and additional strain BC21 in E1, E2, E3 and E5) were produced on potato dextrose agar (PDA; Difco, Detroit, MI, USA) in a growth chamber at 21 °C with a 16-h photoperiod [162 μmol m^−2^ s^−1^ photosynthetic photon fluence rate (PPFR)]. These strains are known from previous work of our group for their high (BC1) and moderate (BC21) aggressiveness on tomato plants (Ajouz *et al.*, 2010). Conidia were collected in sterile distilled water from the surface of 14-d-old cultures and the suspensions were adjusted to 10^6^ conidia mL^−1^. Strain BC1 was applied to the petiole stub of leaf 4 in experiments E1, E2, E3 and E5, and to those of leaves 4 and 6 in E4, E6, E7 and E8. Strain BC21 was applied to leaf 6 in E1, E2, E3 and E5.

Inoculated plants were incubated for 7 d in growth chambers set at 21 °C day/18 °C night, 90 % relative humidity and 14 h of daylight (300 μmol m^−2^ s^−1^ PPFR). In all experiments, the proportionality of water and N supply in the different treatments was maintained during this incubation period. Two daily irrigation events were programmed, with actual evapotranspiration adjusted from drainage of plants receiving full irrigation water. In E1 to E5, only inoculated plants were incubated in the growth chambers. In E6 to E8, both mock- and *Botrytis*-inoculated plants were installed in randomized treatment × cultivar combinations, by groups of five plants. In all the experiments, each combination of treatment × genotype × sampling date was observed in five biological replicates.

Disease development was monitored on the petiole stubs and the stems. Lesion lengths on stems were recorded daily from day 3 to day 7 post-inoculation (DPI) in E1 to E5, and the area under the disease progress curve (AUDPC) was calculated as in Lecompte *et al.* (2010). In E6 to E8, lesion lengths were recorded at 7 DPI.

### Inoculation of detached leaves with mycelium of *B. cinerea*

In four experiments, the leaves removed from the plants for the inoculation of pruning wounds were used to carry out tests on the lamina tissue. In experiments E3 and E5, three leaf discs (3 cm in diameter) were excised from leaves 4 and 6 of five tomato plants at 0 DPI, and inoculated with strains BC1 and BC21. In E7 and E8, terminal leaflets were collected, on five plants, from leaves 8 and 9 at 0, 3 and 7 DPI, and inoculated with strain BC1. All leaf segments were placed, adaxial side upward, on wet filter paper in clear PVC boxes and immediately inoculated with mycelial discs of the pathogen. The mycelial discs, 3 mm in diameter, were excised from the growing margin of 3-d-old colonies of *B. cinerea* grown on PDA at 21 °C. Leaves were incubated in the same growth chamber as described above, and disease severity was assessed 48 h after inoculation by measuring the area of the lesions. In all experiments, each combination of treatment × genotype × sampling date was observed in five or six biological replicates.

### Biochemical analysis of plant tissue

Batches of five plants per treatment × genotype combination were reserved for tissue analyses. In E1 to E5, samples of tissues were collected from these batches on the same day when the other plants were inoculated. Entire leaves (petiole and lamina) and stem segments were collected separately. Leaves 3 to 6 were collected, as well as stem segments (4 cm long) around the insertion of leaves 3 and 5. In experiments E6 and E7, disease progress and tissue contents were measured on the same individuals. Samples were obtained at 3 and 7 DPI from *Botrytis*-inoculated and mock-inoculated plants placed in growth chambers at 0 DPI. Leaves 3, 5 and 7 were collected, as well as stem segments between leaves 3 and 7. A 2-cm-long sample of symptomless stem tissue was collected on each side of the lesions caused by *B. cinerea*, at a distance of 1 cm from the lesion margins. The collected samples were immediately frozen in liquid nitrogen and kept at − 80 °C before analysis.

Harvested leaves and stems were crushed and lyophilized. Soluble sugars (glucose, fructose and sucrose) and organic acids (citric acid, malic acid and quinic acid) were extracted according to the method described by Gomez *et al.* (2002) and analysed by HPLC (Waters 410, Part WAT070390, Milford, USA). Starch content was assessed in the supernatant after a hydrolysis step. The glucose released by starch hydrolysis was quantified using the micro-method described in Gomez *et al.* (2007) and starch content was calculated. Nitrogen and carbon contents were measured according to the Dumas method with an element auto-analyser (Flash EA 1112 series; Thermo Fisher Scientific, Courtaboeuf, France). Nitrate content was determined in water extracts of the dried material with an auto-analyser (AQUATEC 5500; Tecator, Hoganas, Sweden) using a colorimetric assay to measure nitrite after nitrate reduction by cadmium. Amino acids (in E1 to E5) were assessed by HPLC, using a AccQ Tag amino acid analysis column (WAT052885l; Waters) after derivatization with 6-aminoquinolyl-*N*-hydroxysuccinimidyl carbamate.

### RNA extraction and quantitative real-time PCR

Quantitative real-time PCR (qRT-PCR) analyses were performed on stem tissues from ‘Momor’ in E7. Total RNA was isolated from 50 mg of lyophilized powder using the commercial kit Tri Reagent solution (AM9738; Ambion, Applied Biosystems, Carlsbad, CA, USA) as described by the manufacturer. Then, a DNase treatment was applied with a TurboDNA-free kit (AM1907; Ambion, Life Technologies). Total RNA was quantified in a spectrophotometer (Nanovue, GE Healhcare, Life Sciences, Velizy-Villacoublay, France) and RNA quality was verified, using 0·8 % agarose gel electrophoresis. Then, 100 ng of total RNA was used for cDNA synthesis, using oligo-(dT)_18_ anchor primer and the Reverse Transcriptase Core Kit (Eurogentec, Angers, France) according to the manufacturer’s instructions. qRT-PCR amplification was performed with the Takyon No Rox SYBR Core kit dTTP blue (Eurogentec) on a Mastercycler ep realplex cycler (Eppendorf, Hamburg, Germany). PCRs were performed in a 96-well plate, using a three-fold cDNA dilution in triplicate as template, with 200 nm forward and reverse primers. The expression level of a tomato actin gene (gene accession no.: FJ532351) was used as an internal control to normalize the expression data for the target genes PR1a (pathogenesis-related protein 1) and COI1 (coronatine-insensitive protein1). Actin was selected from among five reference genes, because its expression was the most stable in our conditions. Specific primers of PR1a and COI1 are listed in Supplementary Data Table S1. The relative expression levels of target genes were shown as fold changess of the expression level in uninfected and unstressed control plants at 0 DPI (control) using the 2^ΔΔCT^ method: $2_{\text{target},\text{control}}^{(\text{MeanCt}}$^−Ct^_target, sample_^)^/$2_{\text{actin},\text{control}}^{(\text{MeanCt}}$^−Ct^_actin, sample_^)^. Results were transformed to a log_2_ scale.

### ABA quantification

Stem ABA content was quantified on ‘Momor’ in E7. ABA was extracted from 20 mg of lyophilized stem tissue with the Phytodetek ABA Test Kit (PDK, 09347/0096; Agdia, Elkhart, IN, USA). The analysis was performed in a 96-well plate, using a 30-fold extract dilution in triplicate according to the manufacturer’s instructions. An Infinite M200 lecturer (Tecan Trading AG, Männedorf, Switzerland) was used at 405 nm to determine sample concentrations.

## Results

### High nitrogen nutrition decreases the susceptibility of stems and detached leaves

**Fig. 1.**
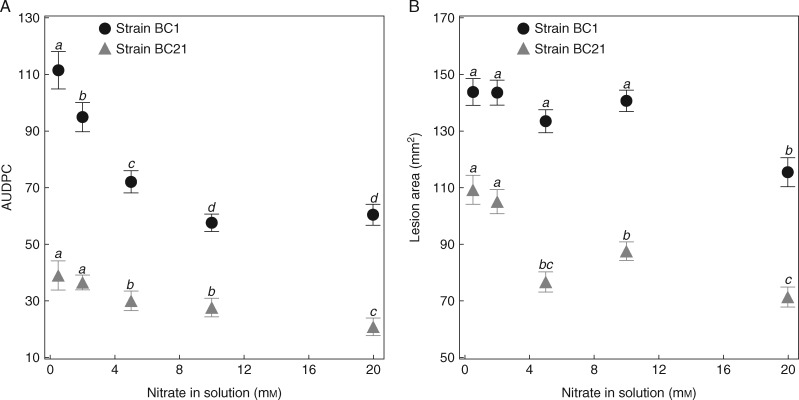
Disease severity caused by *Botrytis cinerea* on tomato stems (A) and detached leaves (B) under various N supply regimes. (A) Area under the disease progress curve (AUDPC) calculated from lesion lengths on tomato stems measured between 3 and 7 days after inoculation with a highly aggressive strain (BC1, black circles; ANOVA for the model AUDPC = Nitrate *f* = 102, *P* < 0·0001, *r*^2^ = 0·39) or a moderately aggressive strain (BC21, grey triangles, ANOVA for the model AUDPC = Nitrate *f* = 37, *P* < 0·0001, *r*^2^ = 0·21). Each observation is the mean of the AUDPC for two infection sites per plant. Each symbol is the mean ± standard error of 25 observations for the highly aggressive strain (corresponding to pooled data from experiments E1 to E5, five plants per nitrate level, one observation per plant) and 20 measurements for the moderately aggressive strain [pooled data from four independent tests (E1, E2, E3, E5), five plants per nitrate level, one observation per plant]. Letters above symbols indicate significant differences between nitrate treatments according to a Student Newman Keuls test, one test per *B. cinerea* strain. (B) Lesion area measured on tomato leaf discs, 2 d after inoculation with a mycelial disc grown on a PDA plate. Each symbol is the mean and standard error of the mean ± standard error of 30 measurements, corresponding to pooled data from two independent experiments, five plants per nitrate level and three leaf discs per plant (BC1, black circles, ANOVA for the model AUDPC = Nitrate *f* = 7·1, *P* < 0·0001, *r*^2^ = 0·06; BC21, grey triangles, ANOVA for the model AUDPC = Nitrate *f* = 18, *P* < 0·0001, *r*^2^ = 0·14). Letters above symbols indicate significant differences between nitrate treatments according to a Student Newman Keuls test, one test per *B. cinerea* strain.

A higher nitrate concentration in the nutrient solution consistently decreased the susceptibility to *B. cinerea* of stem tissues (AUDPC measured between 3 and 7 DPI, Fig. 1A) and leaf discs (lesion area at 2 DPI, Fig. 1B). For both stems and leaves, similar response curves were obtained with strains of the pathogen showing contrasted aggressiveness levels. Lowest disease severities were observed at 10–20 mm${\text{NO}}_{3}^{-}$ in the nutrient solution, 10 mm${\text{NO}}_{3}^{-}$ being the threshold level for maximal plant growth (data not shown). The coefficient of variation of disease severity was lower in leaf assays than in stem assays; fast disease progress in all the bioassays performed on detached leaves may have limited the observation of more contrasted defence responses. Stem bioassays on entire plants were further analysed.

### The relative fructose content at the time of infection is consistently correlated with tomato stem susceptibility to *B. cinerea*

**Fig. 2.**
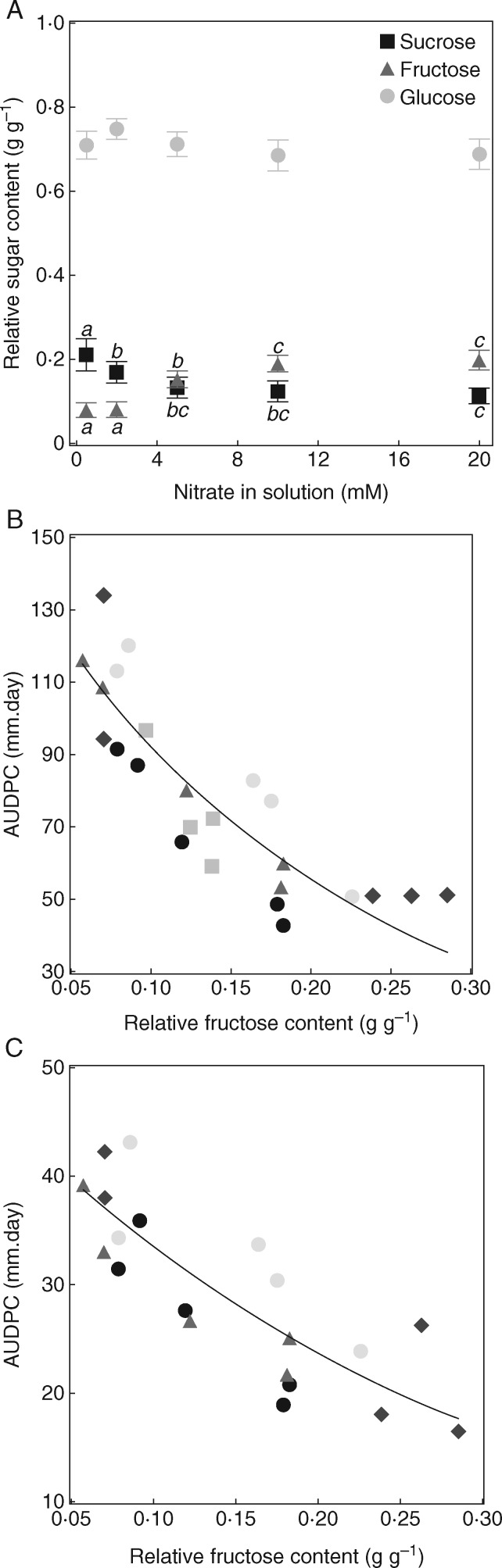
Relationship between disease severity and stem relative sugar contents at the time of infection [0 days post infection (DPI)] for plants grown under various N supply regimes. (A) Relative sucrose [RSC, sucrose/(glucose + fructose+sucrose), black squares], fructose [RFC, fructose/(glucose+fructose+sucrose), grey triangles] and glucose [RGC, glucose/(glucose+fructose+sucrose), light grey circles] contents at 0 DPI in stem tissues of tomato grown at different nitrate supplies. Each symbol is the mean ± standard error of 25 observations, corresponding to pooled data from five independent experiments, with observations on five plants per nitrate level in each experiment. Letters above or below symbols indicate significant differences between nitrate treatments according to a Student Newman Keuls test, one test per sugar. (B, C) Plots of AUDPC versus RFC at 0 DPI for a highly aggressive strain [BC1, (B)] and a moderately aggressive strain [BC21, (C)] of *Botrytis cinerea*, in experiments with contrasted N supply. Each symbol corresponds to the mean of five plants per nitrate treatment, where the relative fructose content is the average RFC at 0 DPI, and the AUDPC the average AUDPC at 7 DPI. Each symbol corresponds to an independent experiment performed with the highly aggressive strain (five experiments E1 to E5), and the moderately aggressive strain (four experiments E1, E2, E3 and E5). The solid lines correspond to exponential regressions [*y*  =  154 × exp(−5·2*x*), *f* = 459, *P*<0·0001, 25 d.f. in B; *y* = 47 × exp(−3·4*x*), *f* = 435, *P*<0·0001, 20 d.f. in C).

In experiments E1 to E5, we examined the relationships between disease severity and stem primary composition at the time of infection. High nitrate supply increased plant total N, nitrate, fructose, as well as individual and total amino acid contents (Supplementary Data Fig. S1). It also reduced the C/N ratio and the starch and sucrose contents, and marginally modified the glucose and organic acid contents (Fig. S1). The relative sucrose content (RSC; sucrose/(glucose + fructose + sucrose)] decreased significantly with nitrate supply (Fig. 2A). By contrast, RFC increased up to 10 mm${\text{NO}}_{3}^{-}$, while the relative glucose content (RGC) did not vary significantly with nitrate supply. Among the absolute contents in the main sugars and acids (either expressed on a dry or a fresh mass basis) or the ratios of contents in those molecules, measured at 0 DPI, RFC best correlated with disease severity caused by either highly aggressive (Fig. 2B) or moderately aggressive (Fig. 2C) strains of *B. cinerea*. This relationship was consistent among the different experiments. The severity of disease also correlated with the total N content, C/N ratio and RSC, but the *F* statistics obtained in various linear or non-linear regressions were consistently lower than those with RFC (Supplementary Data Table S2). No consistent correlations were observed between disease severity and hexoses (glucose + fructose), soluble sugars (glucose + fructose + sucrose) or total carbohydrates (starch + soluble sugars).

### Evolution of stem sugar content after infection of plants grown at various N supply

**Fig. 3.**
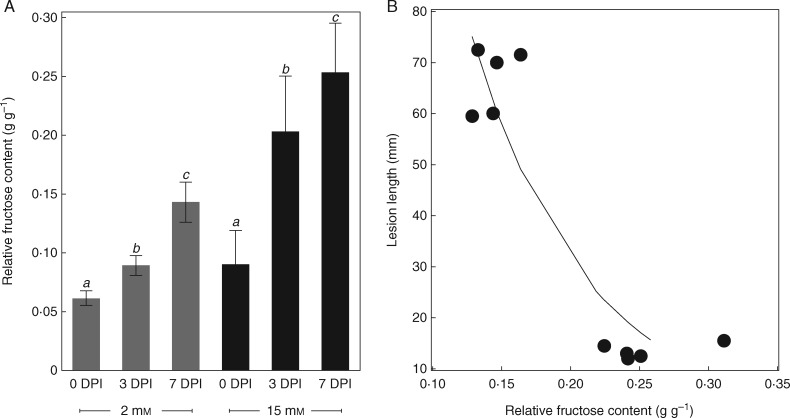
Evolution of the relative fructose content [RFC, fructose/(glucose+fructose+sucrose)] after infection and regression between RFC and disease severity at 7 days post-infection (DPI) for plants grown under different N supply regimes. Data are from experiment E6 on ‘Momor’. (A) RFC of tomato stems during the course of infection, in plants grown at 2 or 15 mm${\text{NO}}_{3}^{-}$ in the nutrient solution. Each bar is the mean ± standard deviation of observations on five plants. Letters above the bars indicate significant differences between sampling dates according to a Student Newman Keuls test, one test per N treatment. (B) Plot of lesion length versus RFC. Lesion length on tomato stems measured 7 DPI of a highly aggressive strain (BC1) on petiole wounds of leaves 4 and 6. Each observation is the mean of the lesion length around the two infected petioles. Each symbol corresponds to one plant. The RFC and lesion lengths were measured on the same plants at 7 DPI. The solid line corresponds to an exponential regression [*y* = 357 × exp(−12·1*x*), *f* = 82, *P* < 0·0001, 10 d.f.].

The evolution of stem sugar contents during the course of infection (at 0, 3 and 7 DPI) was monitored in another experiment (E6) with two contrasted nitrate regimes. In this experiment, in concordance with previous ones, lesion lengths at 7 DPI decreased from 66 mm at 2 mm${\text{NO}}_{3}^{-}$ in the nutrient solution to 13 mm at 15 mm${\text{NO}}_{3}^{-}$ (*P* < 0·0001). In contrast to previous experiments, all the soluble sugar contents at 0 DPI were lower at higher N supply, possibly as a consequence of lower natural radiation in this experiment (Supplementary Data Fig. S2). As in experiments E1–E5, the mean RFC at 0 DPI was higher at high N supply (*P* = 0·02). In comparison with mock-inoculated plants, stem infection by *B. cinerea* induced, at low nitrate supply, an increase in the fructose content (+95 %) and a limited decrease in the sucrose content (−33 %), while at high nitrate supply, no differences were found (Fig. S2). Although the increase in the absolute stem fructose content was higher at low N, the RFC increase after inoculation was greater at high N (Fig. 3A). At 7 DPI, RFC was strongly correlated with lesion length (Fig. 3B)

### Contrasted effects of water supply on the susceptibility of stems and leaves to *B. cinerea*

**Fig. 4.**
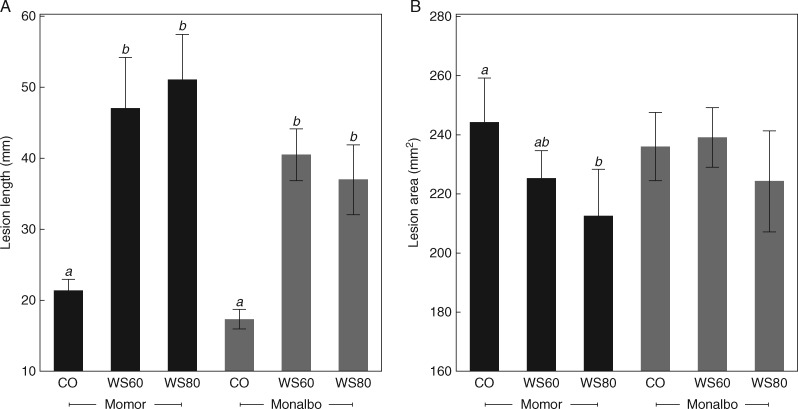
Disease severity caused by *Botrytis cinerea* on tomato stems (A) and detached leaves (B) under various water supply regimes. Data are from experiment E7. (A) Lesion length on tomato stems measured 7 days after inoculation of a highly aggressive strain (BC1) on petiole wounds of leaves 4 and 6. Each observation is the mean of the lesion length around the two infected petioles. CO: fully watered control plants; WS60: irrigation deficit of − 60 %; WS80: irrigation deficit of − 80 %. Each bar is the mean ± standard deviation of observations on five plants. Letters above the bars indicate significant differences between water treatments according to a Student Newman Keuls test, one test per cultivar. The test was repeated once with similar results. (B) Lesion area measured on tomato leaflets, 2 d after inoculation with a mycelial disc excised from a colony grown on PDA medium. CO: fully watered control plants; WS60: deficit irrigation of − 60 %; WS80: deficit irrigation of − 80 %. Each bar is the mean ± standard deviation of 25 observations per water treatment, on five leaflets of the 8^th^ and 9^th^ leaves sampled on five plants at 0 DPI, and ten leaflets sampled on ten plants at 3 and 7 DPI (five plants infected on petiole wounds and five plants mock-inoculated). Observations were pooled because no statistical differences were found between sampling dates or between infected and uninfected plants. Letters above the bars indicate significant differences between water treatments according to a Student Newman Keuls test, one test per cultivar. The test was repeated once (experiment E8) with similar results.

Mild or severe water deficits strongly increased lesion size on stems at 7 DPI, as compared to fully watered controls (Fig. 4A). The phenomenon was consistent for the two tomato genotypes tested in experiments E7 and E8. On detached leaves, however, water stress decreased (‘Momor’) or did not significantly affect (‘Monalbo’) susceptibility (Fig. 4B). As in the N tests, the strongest variations were observed on stem bioassays, which were retained for further analysis.

### Evolution of stem sugar content after infection of plants grown at various water supply

In the water supply experiment E7, the sugar stem contents in the dry matter at the time of inoculation (0 DPI) were higher at the highest level of water stress (WS80) (Supplementary Data Table S3). However, since water stress induced a proportional increase in the three sugars, this did not create differences in relative sugar contents between treatments at 0 DPI (Table 1). Infection by *B. cinerea* affected the sugar contents differently depending on the water supply, as follows.

**Table 1:** Relative sugar contents [sucrose/(glucose+fructose+sucrose), glucose/(glucose+fructose+sucrose), fructose/(glucose+fructose+sucrose)] of tomato stem tissues in plants infected by *Botrytis cinerea* (I) and in mock-inoculated control plants (NI), at 0 days post-infection (DPI), 3 DPI and 7 DPI, for two cultivars (‘Momor’ and ‘Monalbo’), grown under various water supply regimes (CO: fully watered control plants; WS60: irrigation deficit of − 60 %; WS80: irrigation deficit of − 80 %)

			C0				WS-60				WS-80			
			0 DPI	3 DPI	7 DPI	Date effect	0 DPI	3 DPI	7 DPI	Date effect	0 DPI	3 DPI	7 DPI	Date effect
‘Momor’	Relative sucrose content	I	0·31	0·24	0·2	*a, b, c*	0·32	0·33	0·25	*a, a, b*	0·3	0·37	0·32	*ns*
		NI	0·31	0·28	0·23	*a, ab, b*	0·32	0·32	0·36	*ns*	0·3	0·41	0·4	*a, a, b*
		Infection effect		*ns*	*			*ns*	***			*ns*	*	
	Relative fructose content	I	0·09	0·08	0·13	*a, a, b*	0·09	0·1	0·11	*a, a, b*	0·09	0·09	0·1	*ns*
		NI	0·09	0·08	0·1	*ns*	0·09	0·08	0·11	*a, a, b*	0·09	0·09	0·1	*ns*
		Infection effect		*ns*	***			*	*ns*			*ns*	*ns*	
	Relative glucose content	I	0·6	0·68	0·67	*a, b, b*	0·59	0·57	0·64	*a, a, b*	0·61	0·53	0·58	*ns*
		NI	0·6	0·63	0·67	*a, ab, b*	0·59	0·6	0·53	*a, a, b*	0·61	0·5	0·49	*a, b, b*
		Infection effect		*ns*	*ns*			*ns*	*			*ns*	*	
‘Monalbo’	Relative sucrose content	I	0·37	0·31	0·24	*a, a, b*	0·39	0·42	0·33	*a, a, b*	0·4	0·42	0·34	*a, a, b*
		NI	0·37	0·3	0·27	*a, b, b*	0·39	0·39	0·42	*ns*	0·4	0·45	0·43	*ns*
		Infection effect		*ns*	*			*ns*	*			*ns*	*	
	Relative fructose content	I	0·08	0·08	0·15	*a, a, b*	0·08	0·1	0·13	*a, a, b*	0·08	0·09	0·12	*a, b, c*
		NI	0·08	0·09	0·11	*a, a, b*	0·08	0·08	0·12	*a, a,b*	0·08	0·07	0·1	*a, a, b*
		Infection effect		*ns*	***			*	*ns*			*	*ns*	
	Relative glucose content	I	0·55	0·61	0·61	*ns*	0·52	0·48	0·55	*ab, a, b*	0·52	0·49	0·53	*ns*
		NI	0·55	0·61	0·62	*a, b, b*	0·52	0·54	0·45	*a, a, b*	0·52	0·48	0·47	*ns*
		Infection effect		*ns*	*ns*			*ns*	*			*ns*	*	

Each value is the mean of five observations. Letters indicate significant differences between sampling dates, according to a Student Newman Keuls test. Asterisks indicate significant differences between *Botrytis*-inoculated and mock-inoculated plants, according to a Student Newman Keuls test (*P < 0·05, ***P < 0·001, ns: not significant).

#### Sucrose.

At full water supply (CO), the sucrose contents of both *Botrytis*- and mock-inoculated plants decreased after inoculation, while no differences were found for plants under water stress (Table S3). Infection by *B. cinerea* also reduced the proportion of sucrose in the sugar pool. The RSC of infected plants was significantly lower at 7 DPI than that of mock-inoculated plants, regardless of the water treatment and genotype (Table 1).

#### Glucose.

Regardless of the irrigation regime, the glucose content of mock-inoculated plants decreased between 0 and 7 DPI. In plants infected by *B. cinerea*, the glucose content also decreased in the CO treatment, but did not change significantly under water stress (Table S3). As a consequence, a significantly higher RGC was found at 7 DPI in infected than in mock-inoculated plants produced under water stress (Table 1). By contrast, no effect of infection on RGC was found in fully watered plants at 7 DPI (Table 1). On average across water supply treatments, glucose decreased by 22 % in mock-inoculated plants, and by 4 % in *Botrytis-*inoculated plants.

#### Fructose.

**Fig. 5.**
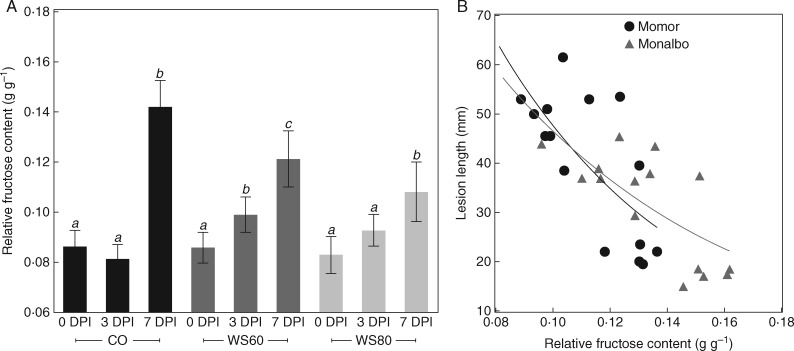
Evolution of relative fructose content (RFC) after infection and regression between RFC and disease intensity at 7 days post-infection (DPI) for plants grown under different water supply regimes. Data are from experiment E7 on ‘Momor’ and ‘Monalbo’. (A) RFC of tomato stems during the course of infection; CO: fully watered control plants; WS60: deficit irrigation of − 60 %; WS80: deficit irrigation of − 80 %. Each bar is the mean ± standard deviation of observations on five plants. Letters above the bars indicate significant differences between sampling dates according to a Student Newman Keuls test, one test per water treatment. (B) Plot of lesion length versus RFC. Lesion length on tomato stems measured 7 d after inoculation of a highly aggressive strain (BC1) on petiole wounds of leaves 4 and 6. Each observation is the mean of the lesion length around the two infected petioles. Each symbol corresponds to one plant. RFC and lesion lengths were measured on the same plants at 7 DPI. The solid line corresponds to exponential regressions [*y* = 226 × exp(−15·6*x*), *f* = 109, *P* < 0·0001, 15 d.f. for ‘Momor’ and *y* = 154 × exp(−11·9*x*), *f* = 123, *P* < 0·0001, 15 d.f. for ‘Monalbo’).

The fructose content of mock-inoculated plants did not vary significantly after inoculation between 0 and 7 DPI. By contrast, the fructose content of plants inoculated with *B. cinerea* increased significantly in ‘Monalbo’ in all water treatments (+29, +62 and +48 % in CO, WS60 and WS80, respectively), and in CO plants in ‘Momor’ only (+14, +37 and +1 % in CO, WS60 and WS80, respectively) (Table S3). Overall across water treatments and genotypes the fructose content increased by 32 % in *Botrytis-*infected plants, as compared to 7 % in mock-inoculated plants. The RFC of infected plants increased by 66 % in CO, 34 % in WS60 and 27 % in WS80 (Table 1, Fig. 5A with averaged data across genotypes). Despite the comparable increase in fructose content after infection in well-watered and water-stressed plants, the increase in RFC in water-stressed plants was low and not significant; at 7 DPI no differences were found between *Botrytis-* and mock inoculated plants (Table 1). Contrastingly, the RFC of infected plants in CO rose sharply between 3 and 7 DPI; at 7 DPI it was significantly higher in infected than in mock-inoculated plants (Table 1).

Overall, these data show that an increase in RFC, associated with lowered plant susceptibility in well-watered plants, was hampered by glucose accumulation in tissues at reduced water supply. These evolutions of relative sugar contents were strikingly similar for the two genotypes of tomatoes. The RFC at 7 DPI in stems of infected plants was highly correlated to lesion length, for both genotypes (Fig. 5B). A covariance analysis on lesion length at 7 DPI did not reveal an RFC × genotype interaction (*f* = 0·4). Linear or non-linear regressions between lesion length and RGC or RSC at 7 DPI were less significant than that between lesion length and RFC, and revealed RGC × genotype and RSC × genotype interactions (data not shown). The total carbohydrate content (starch + sugars) was not modified significantly, following stem infection by *B. cinerea*, and it was not correlated with disease severity at any date of sampling. A positive correlation between total sugar content (glucose + fructose + sucrose) and disease severity was found, but because the total sugar content during the course of infection was not different in *Botrytis*- and in mock-inoculated plants (Table S3), this correlation appears circumstantial.

### Water availability affects hormonal crosstalk after infection by *B. cinerea*

**Fig. 6.**
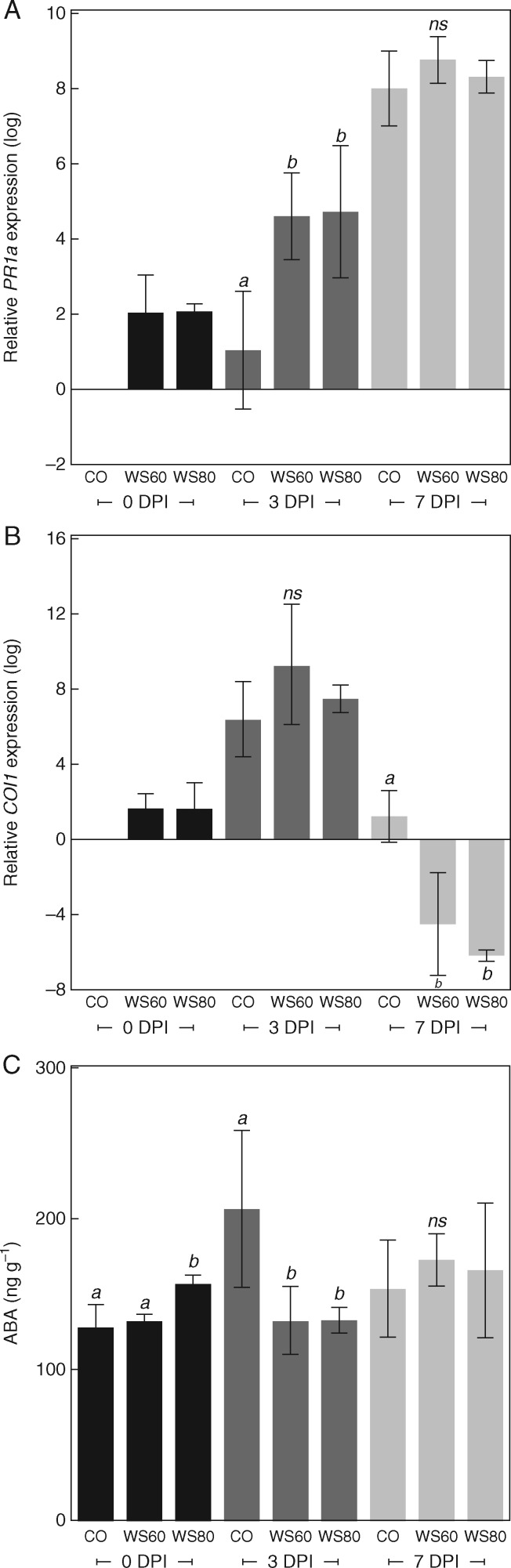
Hormonal signals in tomato (‘Momor’) stem tissues during the course of infection by *Botrytis cinerea*. (A, B) Relative gene expression of *PR1a* (A) and *COI1* (B) at 0, 3 and 7 DPI. CO: well-watered control plant; WS60: deficit irrigation of − 60 %; WS80: deficit irrigation of − 80 %. Data are normalized and expressed as the log_2_ ratio of the expression at 0 DPI of the well-watered control plant. Each bar is the mean ± standard deviation of three biological replicates corresponding each to the mean of three measurements on one plant. Letters above the bars indicate significant differences between water treatments according to a Student Newman Keuls test, one test per sampling date. (C) ABA content of stem tissues, treatments as in (A) and (B). Each bar is the mean ± standard deviation of three observations corresponding each to the mean of three biological replicates corresponding each to the mean of three measurements on one plant. Letters above the bars indicate significant differences between water treatments according to a Student Newman Keuls test, one test per sampling date. ns: not significant.

Markers for SA (*PR1a*) and JA (*COI1*) defence signalling, as well as the ABA contents of stems were monitored after inoculation in E7 in ‘Momor’. Inoculation with *B. cinerea* induced, in comparison with mock-inoculated plants, a higher expression of *PR1a* at 3 and 7 DPI (Supplementary Data Fig. S3A, B). Also, upon infection by *B. cinerea*, *PR1a* expression was significantly higher in water-stressed than in fully watered plants, at 3 DPI but not at 7 DPI (Fig. 6A). *COI1* expression increased greatly between 0 and 3 DPI, both in *Botrytis*- and in mock-inoculated plants (Fig. 6B, Supplementary Data Fig. S3C). At 7 DPI, *COI1* expression was significantly lower in *Botrytis*- than in mock-inoculated plants. Among *Botrytis*-inoculated plants, it was significantly lower for plants produced under water deficit than for CO plants (Fig. 6B). Higher *COI1* transcript levels in mock-inoculated plants (Supplementary Data Fig. S3D) suggested that the late repression of *COI1* was a response to pathogen infection. At 7 DPI, *COI1* expression in *Botrytis*-inoculated plants was linearly correlated with RFC (*P* < 0·0001, *r*^2^ = 0·85, 15 d.f.). The ABA content of mock-inoculated plants at 0 and 3 DPI was higher in the WS80 treatment and, at 7 DPI, higher in the WS60 and WS80 treatments (Fig. 6C). Interestingly, in infected plants at 3 DPI, a strong peak of ABA was observed in CO plants (Fig. 6C). In this control irrigation treatment, because the ABA content was significantly lower in mock- than in *Botrytis*-inoculated plants (Supplementary Data Fig. S3E), this peak was supposedly a response to infection. At 7 DPI, no effect of infection or water supply on ABA content was found (Fig. 6C). As effective defence against necrotrophic fungi is believed to rely on JA signalling, the hormonal patterns observed in this study, showing higher repression of JA in water-stressed plants, coincide with their higher susceptibility to *B. cinerea*.

## DISCUSSION

The abiotic environment is known to affect the susceptibility of plants to pathogens (Atkinson and Urwin, 2012). In this study we performed multiple tests on tomato leaves and stems challenged by *B. cinerea*, under a range of nitrogen and water availabilities. Experiments with various N supply levels confirmed earlier results showing a decreased susceptibility of tomato leaves and stems to *B. cinerea* at higher N supply (Hoffland *et al.*, 1999; Lecompte *et al.*, 2010). Water shortage slightly decreased the severity of symptoms caused by *B. cinerea* on detached leaves, on one of the two tomato genotypes tested. A lower susceptibility to *B. cinerea* of tomato leaves under water stress was also reported by Achuo *et al.* (2006). In the present study, however, we found opposite results on stems, with a much greater lesion size under reduced water supply than in the control. These results emphasize the potentially specific effects of abiotic and biotic interactions depending on the organ and intensity of stress considered (Atkinson and Urwin, 2012). In the present study on tomato stems, RFC was identified as a strong marker of susceptibility of tomato to *B. cinerea*: (1) in a range of abiotic environments and for several genotypes, a higher RFC at the time of infection was correlated with lower susceptibility; (2) in all the infection reactions examined, the RFC in stem tissues around the fungal lesion was stable or increased, as a result of a null to positive accumulation of fructose and, depending of the abiotic environment, variable evolutions of sucrose and glucose; and (3) the increase in RFC after inoculation with *B. cinerea* was inversely proportional to disease severity at 7 DPI. No other combinations of the soluble sugars or total carbohydrates allowed similar conclusions. It could not be excluded that some of the sugar ratios observed in the present study may be unfavourable for intrinsic fungal growth, hampering successful fungal colonization as a result of trophic limitation or reduced virulence. However, *B. cinerea* has been shown to grow similarly well *in vitro* on media with various hexose compositions (Edlich *et al.*, 1989) and necrotrophic fungi possess specific sugar transporters for both hexoses, including a specific transporter of fructose (Doehlemann *et al.*, 2005). Most probably, a specific RFC level favoured defence, as constitutive RFC levels which allowed minimal fungal growth after infection (experiments E1 to E5) were comparable with those attained by the plant after infection (experiments E6 and E7), suggesting a regulation of optimal host fructose status of plant origin.

The sugar status of tomato stems following infection by *B. cinerea* has not, to our knowledge, been described in detail before. Berger *et al.* (2004) showed that in leaves of tomato plants infected by *B. cinerea*, photosynthesis was repressed and the sucrose/hexose ratio started to decrease quickly after infection. In the work presented here, RSC paralleled the sucrose/hexose ratio (Pearson correlation coefficient of 0·99, data not shown). Stems, as sink tissues, contained a much larger amount of sucrose than leaves, and infection by *B. cinerea* did not systematically reduce the absolute or relative sucrose contents of tissues around growing lesions. Under our conditions, both water and nutrient shortages induced an accumulation of sucrose in stems when compared to full water and N supply. Under the hypothesis that stem tissues close to growing lesions were supplied with sucrose from surrounding source leaves, the sucrose status before and after infection appeared to be mainly driven by the abiotic environment. This observation is compatible with the hypothesis that in this necrotrophic interaction between *B. cinerea* and stem tissues, neither fungal growth nor plant defence was limited by soluble sugars. A negative correlation between total leaf carbohydrates and disease caused by *C. hingginsianum* on Arabidopsis has been observed (Engelsdorf *et al.*, 2013). The gene encoding for the Arabidopsis sugar transport protein STP13 is induced upon infection by *B. cinerea*, suggesting that cellular glucose uptake might supply the energy required for defence (Lemonnier *et al.*, 2014). These results support the hypothesis that high sugar levels and turnover in leaves foster resistance to certain hemibiotrophs and necrotrophs. Here, stem infection by *B. cinerea* induced differential glucose and fructose accumulation. In comparison with mock-inoculated plants, infected plants slightly decreased, maintained or increased their glucose content. *Botrytis cinerea* has been shown to manipulate the host’s hormonal defence signalization by way of certain effectors, favouring SA expression (El Oirdi *et al.*, 2011). In the experiment with various water supplies, a higher expression of the SA marker PR1a was associated with increased stem glucose contents in infected tissues. As glucose accumulation under mock-inoculation was not observed whatever the abiotic environment, and because glucose content did not increase in less susceptible plants, this increase in glucose content appeared favourable to virulence. Since glucose content and the expression level of an SA marker increased concurrently in situations of high susceptibility, a dependency link between the two phenomena could not been excluded. Also, it cannot be excluded from the results presented in this study that the accumulation of glucose in host tissues is the result of a fungal manipulation of the host metabolic status. On the other hand, a high fructose accumulation in stem tissues was not necessary for reduced susceptibility, as optimal RFC adjustments could be observed concurrently with only limited increases in fructose content. Consistent with this finding, we previously observed on lettuce leaves that high fructose contents and an RFC above 30 % at the time of inoculation were related to susceptibility to *B. cinerea* and *S. sclerotiorum* (Lecompte *et al.*, 2013). Although invertases and sucrose synthase (SUSY) activities were not measured in the present study, their role in specific adjustments of glucose and fructose contents is likely. Because invertases cleave sucrose into equivalent amounts of glucose and fructose, a role for SUSY, which cleaves sucrose into fructose and UDP-glucose, in the adjustment of RFC can be hypothesized. Alternatively, differential use of fructose and glucose in the metabolism of *Botrytis*-challenged tissues, implying variable hexokinase and fructokinase activities, might be conceivable.

ABA is a known regulator of several physiological processes in response to abiotic and biotic stresses, and a key player in plant hormonal response following *B. cinerea* challenge (Seifi *et al.*, 2013). Some strains of *B. cinerea* possess a functional ABA biosynthetic pathway (Siewers *et al.*, 2006). In the present study, we found slightly higher ABA concentrations in stems at the highest water restriction level at 0 DPI. Interestingly, we observed in the distal tissues of plants with full water supply a transient accumulation of ABA, at concentrations far above those recorded in water-stressed plants, at 3 DPI. This peak of ABA coincided with lower PR1a expression at 3 DPI, in comparison with infected plants under water stress. A link between the two signals could not be excluded, since ABA is known to antagonize SA signalling (Yasuda *et al.*, 2008). ABA is also known to repress the JA/ET-dependent pathway which is required for defence against necrotrophic fungi (Anderson *et al.*, 2004), while SA and JA signalling pathways are mutually antagonistic (Pieterse *et al.*, 2012). Our observations are compatible with this model: a transient ABA peak in stems of fully watered plants, in response to infection, could have coincided with a lower triggering of SA signalling, and as a consequence a lower repression of JA-based defence during the period of lesion expansion, leading to reduced symptoms. Indeed, the expression of PR1a at 3 DPI and that of COI1 at 7 DPI in tissues surrounding lesions were positively and negatively correlated with symptoms, respectively.

In conclusion, these results show that in tomato stems, the RFC in the sugar pool is adjusted after infection by the necrotrophic fungus *B. cinerea*. An optimal RFC adjustment led to reduced susceptibility and was concomitant with favourable hormonal defence signals. The mechanisms underlying a lower susceptibility to *B. cinerea*, requiring sensing and regulation of optimized fructose equilibrium in the host sugar pool, and/or implying differential roles of glucose and fructose in plant defence, remain to be elucidated. As RFC is a strong marker of susceptibility to *B. cinerea* and possibly other necrotrophs, paying greater attention to constitutive RFCs in breeding programmes and crop management strategies might lead to reduced damage caused by important plant diseases.

## SUPPLEMENTARY DATA

Supplementary data are available online at www.aob.oxfordjournals.org and consist of the following. Figure S1: effect of nitrate supply on the content of some primary components of tomato leaves and stems. Figure S2: evolution of tomato stem sugar contents after infection by *B. cinerea* or mock-inoculation, on plants grown at two nitrate supplies. Figure S3: evolution of tomato stem *PR1A* and *COI1* expression and ABA content after infection by *B. cinerea* or mock inoculation, on plants grown at three water supplies. Table S1: sequences of primer pairs and conditions used for real-time PCR. Table S2: statistics of regressions between AUDPC at 7 DPI by *B. cinerea* and various plant metabolites and related ratios at the time of infection. Table S3: soluble sugars, total soluble sugars and starch contents of tomato stem tissues in plants infected by *B. cinerea* and in mock-inoculated control plants.

## Supplementary Material

Supplementary Data
