# Supplementary material for: Reduced susceptibility of tomato stem to the necrotrophic fungus Botrytis cinerea is associated with a specific adjustment of fructose content in the host sugar pool
Source: Ann Bot. 2017 Jan 8;119(5):931–43. doi: 10.1093/aob/mcw240 (PMC5378192; doi:10.1093/aob/mcw240)
Supplement: Supplementary Data [file mcw240_Supp.zip › aob-16371-s06.docx]

Table S3 Soluble sugars, total soluble sugars (TSS) and starch contents of tomato stem tissues in plants infected by *Botrytis cinerea* (I) and in mock-inoculated control plants (NI), at 0 days post infection (DPI), 3 DPI and 7 DPI, for two cultivars (Momor and Monalbo), grown under various water supply regimes (CO: fully-watered control plants; WS60: irrigation deficit of -60%; WS80: irrigation deficit of -80%). Each value is the mean of five observations. Letters indicate significant differences between sampling dates, according to a Student Newman Keuls test. Symbols identify significant differences between Botrytis-inoculated and mock-inoculated plants, according to a Student Newman Keuls test (*: p<0.05, ***: p<0.001, ns: not significant).
