# Supplementary material for: Reduced susceptibility of tomato stem to the necrotrophic fungus Botrytis cinerea is associated with a specific adjustment of fructose content in the host sugar pool
Source: Ann Bot. 2017 Jan 8;119(5):931–43. doi: 10.1093/aob/mcw240 (PMC5378192; doi:10.1093/aob/mcw240)

Fig. S1 Effect of nitrate supply on the stem (triangles) and leaf (rectangles) dry matter content of primary components of tomato: nitrate (a), C:N ration (b), starch (c), sucrose (d), glucose (e), fructose (f), malic and citric acid (g), total amino acids (h). Each symbol is the mean ± standard error of 25 observations, corresponding to pooled data from five independent experiments, with 5 plants per nitrate level in each experiment.


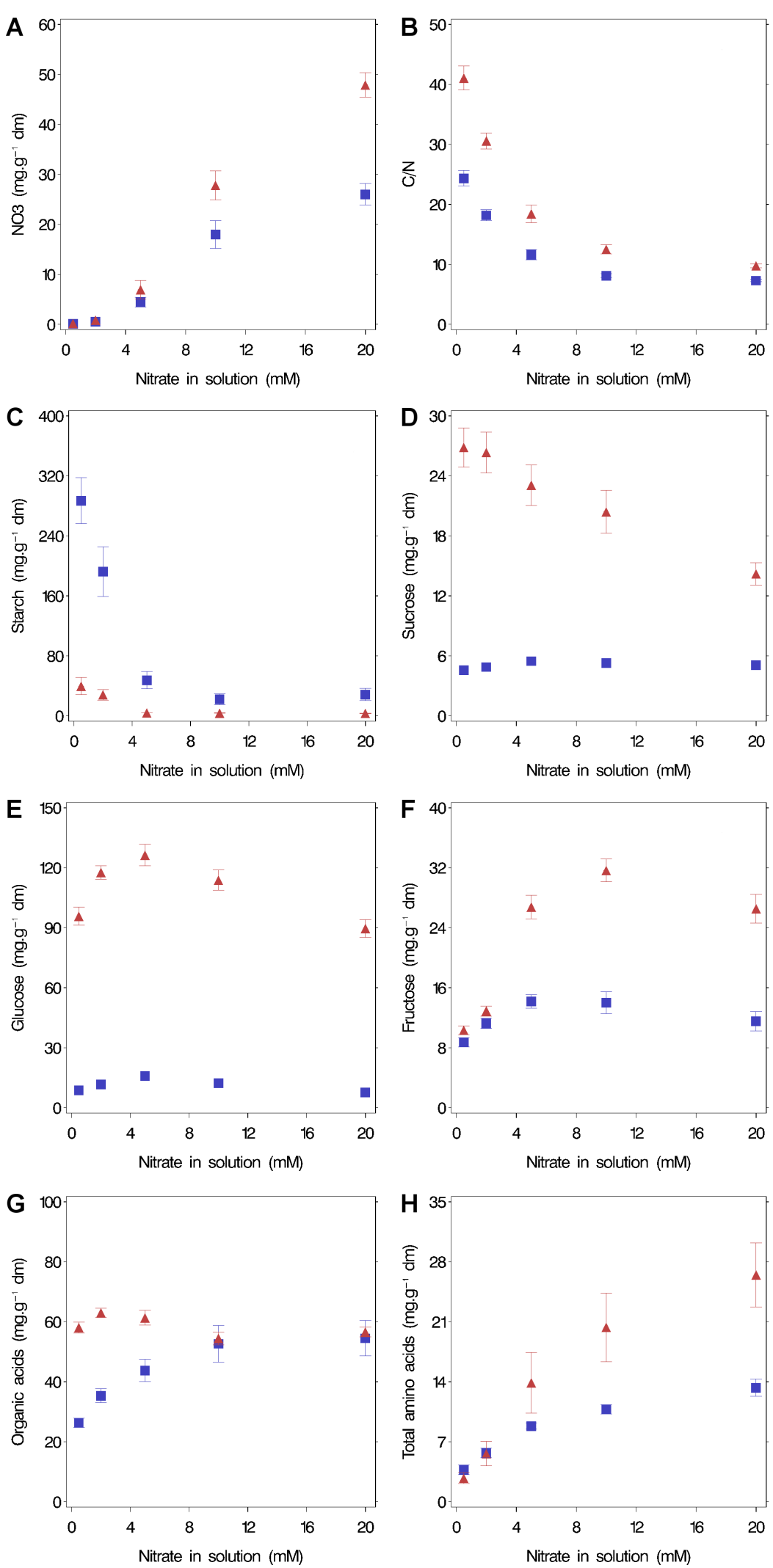

Supplement: Supplementary Data [file mcw240_Supp.zip › aob-16371-s01.docx]
