# Supplementary material for: Reduced susceptibility of tomato stem to the necrotrophic fungus Botrytis cinerea is associated with a specific adjustment of fructose content in the host sugar pool
Source: Ann Bot. 2017 Jan 8;119(5):931–43. doi: 10.1093/aob/mcw240 (PMC5378192; doi:10.1093/aob/mcw240)

Fig. S2 Evolution of tomato stem sugar contents after infection by *Botrytis cinerea* or mock-inoculation, on plants grown at two nitrate supplies, 2 mM NO3- or 15 mM NO3- in the nutrient solution. Each bar is the mean ± standard deviation of 5 observations each on one plant. Letters above the bars indicate significant differences between *Botrytis*-inoculated (I) and mock-inoculated plants (NI) according to a Student Newman Keuls test, one test per sampling date and N treatment.


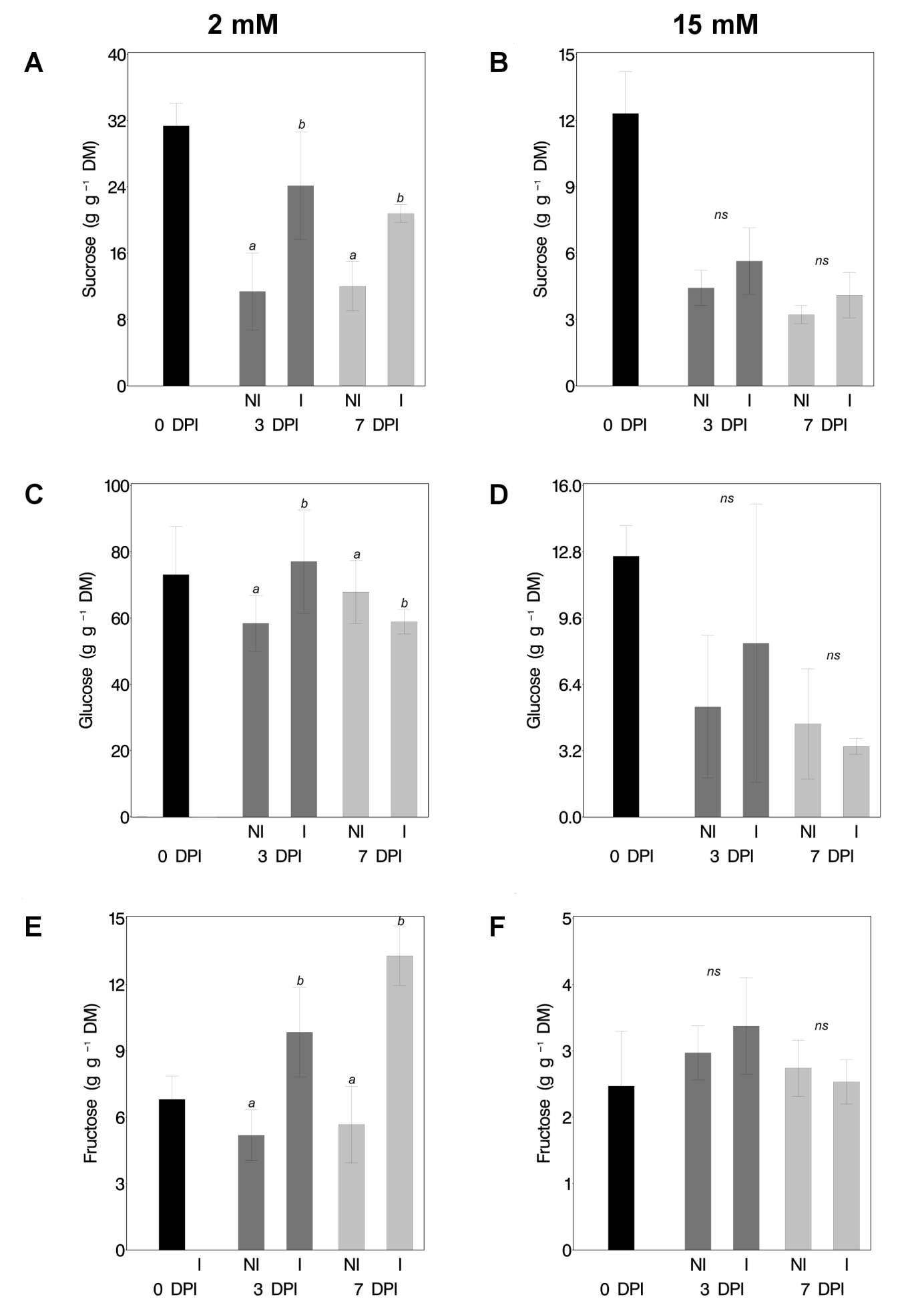

Supplement: Supplementary Data [file mcw240_Supp.zip › aob-16371-s02.docx]
