# Supplementary material for: Reduced susceptibility of tomato stem to the necrotrophic fungus Botrytis cinerea is associated with a specific adjustment of fructose content in the host sugar pool
Source: Ann Bot. 2017 Jan 8;119(5):931–43. doi: 10.1093/aob/mcw240 (PMC5378192; doi:10.1093/aob/mcw240)

Fig. S3 Evolution of tomato stem *PR1A* and *COI1* gene expressions and ABA content after infection by *Botrytis cinerea* or mock inoculation, on plants grown at three water supplies: CO: fully-watered control plants; WS60: irrigation deficit of -60%; WS80: irrigation deficit of -80%. Hormonal markers were measured at 3 and 7 days post infection (3DPI, 7 DPI). Gene expression data are normalized and expressed as the log2 ratio of the expression at 0 DPI of the well-watered and mock-inoculated control plant. Each bar is the mean ± standard deviation of 3 biological replicates, measured each in triplicate. Letters above the bars indicate significant differences between *Botrytis*-inoculated (I) and mock-inoculated plants (NI) according to a Student Newman Keuls test, one test per sampling date and water treatment.


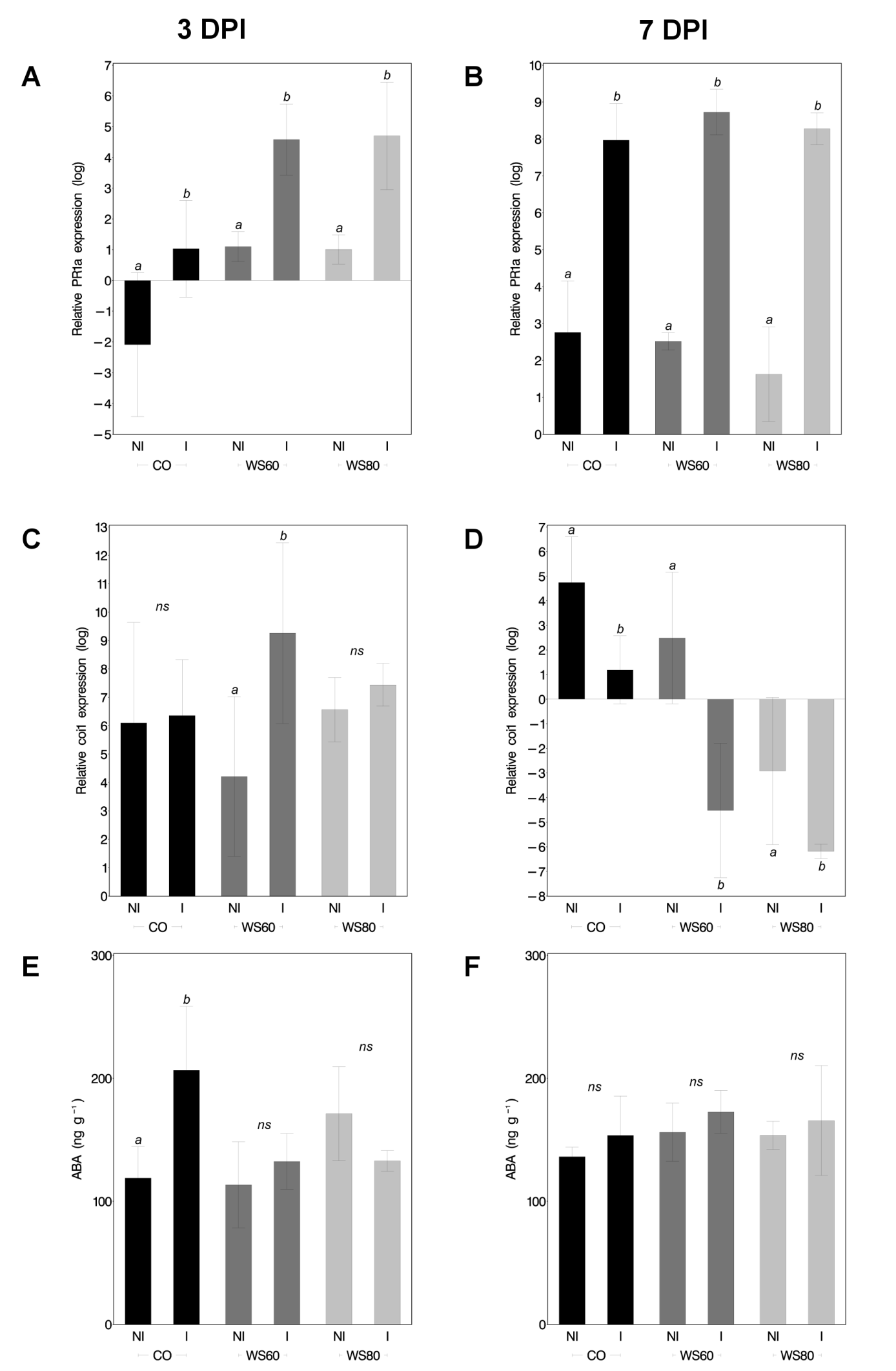

Supplement: Supplementary Data [file mcw240_Supp.zip › aob-16371-s03.docx]
