# Supplementary material for: Reduced susceptibility of tomato stem to the necrotrophic fungus Botrytis cinerea is associated with a specific adjustment of fructose content in the host sugar pool
Source: Ann Bot. 2017 Jan 8;119(5):931–43. doi: 10.1093/aob/mcw240 (PMC5378192; doi:10.1093/aob/mcw240)
Supplement: Supplementary Data [file mcw240_Supp.zip › aob-16371-s04.docx]

Table S1 Sequences of primers pairs and conditions used for real time PCR

| Accession number | Gene | Primer sequence  5’ → 3’ | Amplicon size (bp) | Annealing temperature (°C) | Primer efficiency (%) |
| --- | --- | --- | --- | --- | --- |
| AY423550.1  Nair *et al.* (2015) | Coronatine-insensitive 1 COI1 | Fw: TGCACTTCTTGACACAGCAGCCC  Rev: AGTACTGGCCAAGCACTTCC | 100 | 60 | 98.7 |
| AJ011520 | Pathogenesis-related 1 (PR1a) | Fw:  CGTGCAATTGTGGGTGTC  Rev:  TCTCCAACCCAGTTGCCTA | 195 | 60 | 98.2 |
| FJ532351 | Actine | Fw: ATGACTCAAATCATGTTTGAG  Rev: ACCTTAATCTTCATGCTGCTT | 632 | 60 | 97.2 |
