# Supplementary material for: Reduced susceptibility of tomato stem to the necrotrophic fungus Botrytis cinerea is associated with a specific adjustment of fructose content in the host sugar pool
Source: Ann Bot. 2017 Jan 8;119(5):931–43. doi: 10.1093/aob/mcw240 (PMC5378192; doi:10.1093/aob/mcw240)
Supplement: Supplementary Data [file mcw240_Supp.zip › aob-16371-s05.docx]

Table S2 Statistics of regressions between AUDPC at 7 days post infection (DPI) and various plant stem metabolites and related rations, at the time of infection (0 DPI) by *Botrytis cinerea*. Statistics are presented for lesions caused by a highly aggressive strain (BC1), and a moderately aggressive strain (BC21). Regressions were performed each on 25 observations obtained in five independent experiments. RSC, RFC, RGC: relative sucrose, fructose and glucose contents.

|  |  | **Linear regression** | | | **Exponential regression** | **Hyperbolic regression** |
| --- | --- | --- | --- | --- | --- | --- |
|  | **Variable** | **r2** | **F value** | **p value** | **F value** | **F value** |
|  |  |  |  |  |  |  |
| Highly aggressive strain | Glucose | 0.005 | 0.1 | 0.73 | NC | 101 |
|  | Fructose | 0.58 | 30.9 | <0.0001 | 310 | 320 |
|  | Hexoses | 0.04 | 0.9 | 0.33 | NC | 111 |
|  | Sucrose | 0.13 | 3.4 | 0.07 | 117 | 117 |
|  | RSC | 0.19 | 5.2 | 0.03 | 129 | 116 |
|  | **RFC** | **0.69** | **48.9** | **<0.0001** | **460** | **357** |
|  | RGC | 0.09 | 2.2 | 0.15 | 111 | 112 |
|  | Total sugars | 0.009 | 0.21 | 0.65 | NC | 126 |
|  | Starch | 0.05 | 1.2 | 0.36 | 108 | 101 |
|  | Total carbohydrates | 0.1 | 1.3 | 0.25 | NC | 76 |
|  | Malic acid | 0.0007 | 0.02 | 0.9 | NC | 100 |
|  | Citric acid | 0.28 | 8.9 | 0.007 | 152 | 126 |
|  | Quinic acid | 0.15 | 2.5 | 0.13 | 69 | 62 |
|  | Total acids | 0.017 | 0.24 | 0.63 | NC | 61 |
|  | Nitrate | 0.48 | 20.5 | 0.0002 | 238 | 175 |
|  | Total N | 0.6 | 33 | <0.0001 | 314 | 322 |
|  | C/N | 0.65 | 38 | <0.0001 | 321 | 326 |
|  |  |  |  |  |  |  |
| Moderately aggressive strain | Glucose | 0.03 | 0.7 | 0.42 | NC | 132 |
|  | Fructose | 0.47 | 16.4 | 0.0007 | 269 | 288 |
|  | Hexoses | 0.01 | 0.2 | 0.65 | NC | 128 |
|  | Sucrose | 0.24 | 5.8 | 0.027 | NC | 171 |
|  | RSC | 0.22 | 5.3 | 0.035 | 165 | 157 |
|  | **RFC** | **0.67** | **37.2** | **<0.0001** | **435** | **344** |
|  | RGC | 0.18 | 4.1 | 0.059 | 155 | 325 |
|  | Total sugars | 0.001 | 0.02 | 0.88 | NC | 120 |
|  | Starch | 0.02 | 0.5 | 0.51 | NC | 126 |
|  | Total carbohydrates | 0.1 | 1.3 | 0.25 | NC | 81 |
|  | Malic acid | 0.01 | 0.3 | 0.6 | NC | 130 |
|  | Citric acid | 0.28 | 7 | 0.016 | 180 | 165 |
|  | Quinic acid | 0.08 | 1.2 | 0.28 | 116 | 110 |
|  | Total acids | 0.048 | 0.71 | 0.41 | NC | 116 |
|  | Nitrate | 0.48 | 16.7 | 0.0007 | 279 | 7.8 |
|  | Total N | 0.64 | 32.3 | <0.0001 | 417 | 340 |
|  | C/N | 0.63 | 32 | <0.0001 | 341 | 328 |
